# Feasibility and Acceptability of Diabetes and Hypertension Screening and Diagnosis by Community Health Workers in Rural Lesotho: A Mixed-Methods Pilot Study

**DOI:** 10.5334/aogh.4738

**Published:** 2025-09-12

**Authors:** Thabo Ishmael Lejone, Felix Gerber, Ravi Gupta, Jennifer M. Belus, Thesar Tahirsylaj, Tristan Lee, Giuliana Sanchez-Samaniego, Maurus Kohler, Maria Ines Haldemann, Fabian Raeber, Andrea Williams, Makhebe Khomolishoele, Palesa Mahlatsi, Pauline Mamoroents’ane Sematle, Lucia Motlatsi, Boikano Matjeane, Dave Basler, Kevin Kindler, Pauline Grimm, Martin Rohacek, Alain Amstutz, Niklaus Daniel Labhardt

**Affiliations:** 1 Division of Clinical Epidemiology, Department of Clinical Research, University Hospital Basel, Basel, Switzerland; 2 University of Basel, Basel, Switzerland; 3 Swiss Tropical and Public Health Institute, Allschwil, Switzerland; 4 SolidarMed, Lesotho; 5 Ministry of Health, Lesotho; 6 Faculty of Business, Economics and Informatics, University of Zurich, Switzerland; 7 SolidarMed, Switzerland; 8 Ifakara Health Institute, Ifakara, Tanzania; 9 Oslo Center for Biostatistics and Epidemiology, Oslo University Hospital, University of Oslo, Oslo, Norway; 10 Population Health Sciences, Bristol Medical School, University of Bristol, Bristol, United Kingdom

**Keywords:** community health workers, home-based, screening, hypertension, diabetes, non-communicable chronic diseases

## Abstract

*Introduction:* Across Africa, community health workers (CHWs) have become an important cadre in prevention and care services. Community-based service delivery models largely overlook non-communicable diseases (NCDs). Although Lesotho`s Village health worker program is well established, it currently offers no NCD services. This pilot study assessed the feasibility and acceptability of CHW-led home-based screening and diagnosis for arterial hypertension and diabetes mellitus in rural Lesotho.

*Methods:* This mixed-methods pilot study involved 10 CHWs from 10 rural villages in two districts of Lesotho. From March 2022 to December 2023, the CHWs enrolled and screened all eligible and consenting participants of their villages for hypertension (using automated blood pressure (BP) measurements) and diabetes (using capillary blood glucose measurements) in a door-to-door approach. All participants aged ≥18 years were eligible for hypertension screening; those aged ≥40 years or with a body mass index (BMI) ≥25 kg/m^2^ were eligible for diabetes screening. 10 purposively sampled participants were interviewed with subsequent qualitative thematic analysis.

*Results:* In the 10 villages, CHWs visited a total of 687 households and enrolled 1811 participants (median age 24 years (interquartile range (IQR): 11-25.5 years), 56.5% female, median BMI 23.4kg/m^2^). Among 803 participants eligible for diabetes screening, 788 (98%) were screened. Overall, 28 (3%) had impaired fasting glucose and 42 (5.3%) had diabetes. Among 1091 participants eligible for hypertension screening, 998 (91.5%) were screened, 50 (5%) had high normal BP, and 268 (26.9%) had hypertension. All participants interviewed expressed a high level of acceptance and appreciation for CHW-led screening and diagnosis of diabetes and hypertension.

*Conclusion:* In this pilot study in Lesotho, CHW-led screening and diagnosis of hypertension and diabetes was highly acceptable and feasible, achieving >90% screening coverage. These results support larger-scale studies and encourage further exploration across diverse regions to assess the impact of CHW-led screening and diagnosis for NCDs.

## Introduction

The burden of non-communicable diseases (NCDs) is continuously increasing, leading to a significant rise in deaths and disabilities across Africa [1]. In Africa, NCDs are projected to surpass infectious diseases as the leading cause of morbidity and mortality by 2030 [2]. Arterial hypertension (aHT) and type 2 diabetes mellitus (DM) rank among the most significant contributors [3–5]. By 2025, it is estimated that 1.5 billion people worldwide will have hypertension [6, 7], with 125.5 million residing in Africa [8]. Similarly, the burden of DM is expected to increase by 129% by 2045 [5, 9] with over 95% of cases attributable to DM [10, 11]. aHT and DM are main risk factors for the development of cardiovascular diseases (CVDs), including stroke, myocardial infarction, heart failure, and chronic kidney disease [12–15].

Globally, 46% and 50% of people living with aHT or DM, respectively, are aware of their condition [5, 16–18]. In Africa, the figures are even lower, with awareness of 27% and 40% for aHT and DM. WHO aims to reduce the diagnostic gap for DM and aHT by improving screening and diagnosis. The target is to ensure that 80% of affected individuals are identified by 2030 [19].

Across Africa, Community Health Worker (CHW) programs have been shown to increase access to prevention services, thereby alleviating the burden on overcrowded primary health care facilities, particularly in the context of HIV programs [20–23]. Involvement of CHWs in the prevention and screening for NCDs has shown promise in various settings [24–29]. A recent small study in South Africa evaluated the feasibility and acceptability of aHT screening by CHWs [24]. However, there is limited evidence from Africa on CHWs independently screening and diagnosing aHT and DM at people’s homes using guideline-directed diagnostic algorithms with minimal supervision from healthcare professionals.

Similar to its neighboring countries in Southern Africa, Lesotho experiences a double burden of diseases with a rise in NCDs while communicable diseases, particularly HIV and tuberculosis, remain prevalent. A recent population-based survey in two districts of the country found an adult prevalence of 21.6% and 5.3% for aHT and DM, respectively, with suboptimal treatment and control rates and high rates of undetected end-organ damage [30–32]. In Lesotho, NCD care is only provided at the health facilities. The high prevalence of NCDs and relevant awareness, diagnosis and treatment gaps underscore the need to develop more accessible NCD service models [30–32].

This mixed-methods pilot study, nested within a population-based cohort study, evaluated the feasibility and acceptability of screening and diagnosing aHT and DM by trained CHWs using the ComBaCaL App [33], a tablet-based clinical decision support system, in 10 villages in Lesotho, Southern Africa. The study further determines the number of people identified with these conditions through the screening program.

## Methods

### Design

This was a prospective pilot study conducted as part of the Community-Based Chronic Care Lesotho (ComBaCaL) project, which aims to improve chronic care in rural Lesotho through innovative, community-based health service delivery models (www.combacal.org). In March 2022, a prospective open pilot cohort study was introduced in 10 villages to pilot the ComBaCaL CHW-based NCD care model, with the goal of informing the ComBaCaL prospective main cohort study and testing innovative CHW-led health interventions for chronic care [34]. The study employed a mixed-methods approach, combining quantitative data from CHW-led screening and diagnosis activities with qualitative data from interviews.

In each of the 10 villages, CHWs were elected by their communities and trained and equipped to conduct household visits for screening and diagnosis of aHT and DM. CHWs used the ComBaCaL App, a tablet-based clinical decision support system to document household visits and guide screening and diagnosis processes [35, 36].

### Setting

The study was conducted between 30 March 2022, and 31 December 2023, in 10 rural villages located in the Butha-Buthe and Mokhotlong districts of Lesotho. These districts are situated in the mountainous highlands and are characterized by challenging terrain, limited transport infrastructure, and difficult access to healthcare facilities [37].

Lesotho is a small, landlocked country in Southern Africa with a population of approximately 2.3 million people. Socioeconomic challenges include a high unemployment rate of 22.5% and 32.4% of the population living below the poverty line [38].

Butha-Buthe and Mokhotlong have estimated populations of around 120,000 and 100,000, respectively [38]. There are three physician-led secondary hospitals and 19 nurse-led primary healthcare centers. The health system in Lesotho faces significant resource constraints, including a shortage of medical professionals, with only 20.7 doctors and nurses per 10,000 people, well below the WHO’s recommended threshold for achieving universal health coverage [39]. The integration of lay CHWs into the health system structures has been adopted in Lesotho since 1978 [40, 41]. CHWs are community members chosen during a community gathering (called Pitso) based on mutual trust between them and community leadership. Elected individuals must undergo a standardized training by the Ministry of Health to be certified as Village Health Workers (the term used for CHW in Lesotho).

### Eligibility and recruitment of CHWs

For this study, the recruitment process followed the national standard procedures. The local village chiefs were contacted and informed about the content and objectives of the study. The village chiefs were then asked for verbal consent for the participation of the village. Subsequently, a letter was sent to consenting village chiefs, outlining the required qualifications for a CHW, and requesting their assistance in organizing the process for CHW election. Minimal qualifications for CHWs were at least a secondary school education and the ability to read and write. A village committee, consisting of the village chief and the area councilor, pre-selected three eligible candidates from the village. Thereafter, a community gathering was held for villagers where one candidate among the three was selected.

### Training of CHWs

The elected CHWs received 20-days training in the areas of pathophysiology, screening, diagnosis, and basic management of NCDs, with a particular focus on DM and aHT. The CHWs were introduced to the handling of the ComBaCaL App, including correct documentation of all screening and diagnosis activities. Furthermore, the training included an introduction to the basics of good clinical practice, including informed consent and confidentiality.

The CHWs were provided with the necessary equipment, including tablets, an automated blood pressure machine (Omron M3 Comfort [HEM7131-E] [42]), a glucometer, lancets, sharp containers, informed consent forms, and information pamphlets. In addition to the national stipend of 45 USD per month, the CHWs received 40 USD per month to cover transport reimbursement to the monthly meeting at the nearest health facility, and costs for phone and internet data.

### Eligibility of participants

All consenting participants in the 10 villages identified by the CHW between 30 March 2022 and 31 December 2023 were eligible for inclusion in the pilot cohort. Pilot cohort participants aged ≥18 years were eligible for aHT screening, participants aged ≥40 years or 18–39 years with a body mass index (BMI) ≥25 kg/m^2^ were eligible for DM screening.

### Procedures

The CHWs conducted regular rounds in their villages, visiting all households. The initial round served to enumerate the village households and household members and to enroll consenting villagers into the cohort. First, oral consent from the head of household was sought to enter the household and to enumerate all household members. Second, all household members were informed about the content, risks, and objectives of the study. They were given sufficient time for consideration and the opportunity to ask questions about the study before individual written consent to participate in the cohort was sought. For those under the age of 18, written consent was sought from a legal guardian. If a person is unable to read/write, they provide consent with a thumbprint, accompanied by the signature of a witness who is at least 21 years old. Additionally, the number of absent household members was recorded. The CHW returned to all households with absent members in the following days to complete the enrollment process. For consenting household members, baseline variables, including socio-demographic information, self-reported HIV status, and history of aHT and/or DM, were collected.

In the second round, cohort participants were visited for DM, and in the third round for aHT screening.

### Screening and diagnosis of aHT

BP screening and diagnosis followed the guidelines of the European Society of Cardiology/European Society of Hypertension (ESC/ESH) and the national standard treatment guidelines of Lesotho [5, 43, 44]. CHWs measured blood pressure (BP) using validated automated devices in accordance with a standardized operating procedure. BP measurements were taken while participants were seated after a five-minute rest period without conversation, in accordance with specific posture and pre-measurement conditions, including the avoidance of caffeine, exercise, or smoking 30 minutes before measurement.

During the initial visit, BP was measured on both arms to determine the reference arm, which was defined as the arm with the higher reading. Subsequently, three BP measurements were taken on the reference arm, and the average of the last two was calculated. Participants were classified as not having aHT if their average BP reading was below 140/90 mmHg. If the reading was between 140–179/90–119 mmHg, the measurement was repeated within 14 days. If the follow-up reading remained in this range or above, aHT was diagnosed. If the follow-up reading was between 130–139/85–89 mmHg, the participants had high normal BP. In the event of a BP reading of 180/120 mmHg and above, the measurement was repeated after 30–60 minutes of rest. If the follow-up reading remained above 180/120 mmHg, the diagnosis of aHT was confirmed. Participants with aHT and clinical alarm symptoms, such as severe headache, chest pain, dyspnea, and neurological symptoms, were referred immediately to the nearest health facility.

### Screening and diagnosis for DM

The DM screening and diagnosis process followed international and national standard guidelines [5, 44]. Blood glucose levels were interpreted as fasting if the patient had not eaten or drunk anything else than water 8 hours before measurement; otherwise, the blood glucose measurement was considered as random blood glucose (RBG). If the fasting blood glucose (FBG) level was ≥5.6 mmol/L, or the RBG level ≥7 mmol/L, confirmatory FBG tests were scheduled on a different day with instructions to fast on the day of measurement.

Participants with confirmatory FBG levels of 5.6−6.9 mmol/L were classified as having prediabetes (pre-DM), while FBG levels ≥7 mmol/L were classified as having DM. Individuals diagnosed with DM underwent further testing for glycosylated hemoglobin (HbA1c). Individuals displaying symptoms of uncontrolled DM (polyuria, polydipsia, weight loss) and FBG ≥7.0 mmol/l or RBG ≥11.1 mmol/L were promptly referred to the district hospital.

### Quantitative analysis

Quantitative data were analyzed to describe the feasibility and acceptability of the CHW-led intervention. We assessed feasibility using rates of service uptake, including the proportion of households and individuals participating in screening and diagnosis. We assessed the acceptability based on consent rates at different stages, reflecting community engagement and willingness to participate.

Descriptive statistics were used to summarize categorical variables as absolute and relative frequencies. Continuous variables were described using medians and interquartile ranges (IQR). Quantitative analyses were conducted using STATA version 16.0.

### Qualitative analysis

The qualitative data were collected through semi-structured, audio-recorded interviews with 10 participants who had undergone screening and/or diagnosis by the CHWs. Participants were purposefully selected to represent a diversity of experiences and perceptions. The selection criteria ensured that one participant from each village was included, with guidance provided to CHWs on identifying individuals with varied levels of engagement in the intervention. The interviews were conducted in Sesotho using an open-ended interview guide, which explored participants’ preferences, acceptance, and experiences with CHW-led screening and diagnosis. The recordings were transcribed verbatim and translated into English. Transcripts were analyzed thematically to identify recurring themes and insights into community perceptions of the intervention. TIL, AW, PM, and JB analyzed the transcripts using open coding and identified themes based on the thematic analysis suggested by Braun and Clarke, employing a theoretical approach [45].

## Results

### Study population

**Figure 1:**
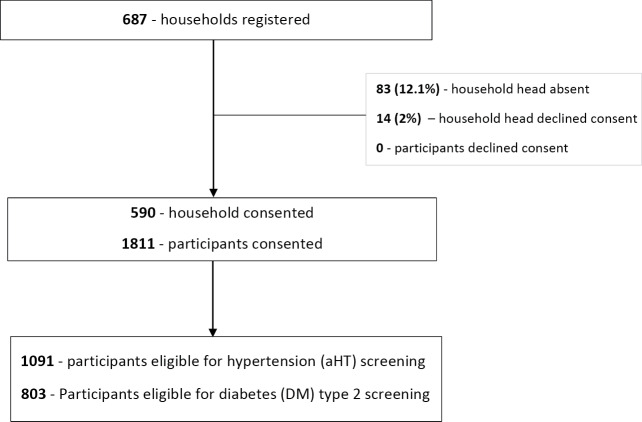
Screened for hypertension and diabetes.

Table 1 summarizes the sociodemographic and clinical characteristics of study participants, while Figure 1 presents an overview of study participation and the screening process. A total of 687 households (HH) were visited by CHWs in the 10 villages. Of these, 590 (85.9%) heads of HH consented to participate, with a total of 1811 consenting household members (participants). The median age of the cohort was 24 years (IQR, 11–25.5), with 1022 (56.5%) of the participants being female. The median BMI was 23.4 kg/m^2^ (IQR, 20.3–27.7). Of the 1811 participants, 1091 (60.2%) met the eligibility criteria for aHT and 803 (44.3%) for DM screening.

### The DM and aHT screening cascade

**Figure 2:**
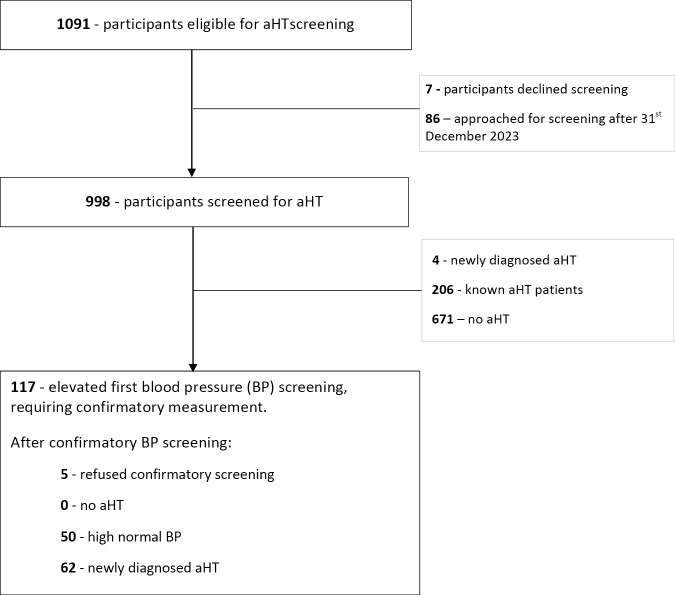
Screened and diagnosed for hypertension.

**Figure 3:**
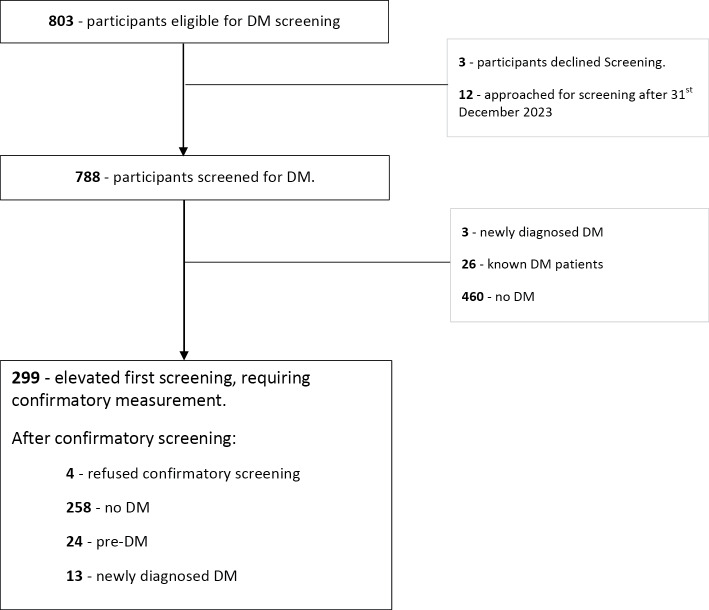
Screened and diagnosed for diabetes (DM).

Figures 2 and 3 present an overview of the study, outlining the screening and diagnosis processes for DM and aHT.

### Screening and diagnosis of aHT

Of the 1091 participants who were eligible, 1005 (92.1%) were offered aHT screening between 30 March 2022 and 31 December 2023, the closure date for this analysis. 86 (7.9%) were offered screening after the closure date. Among the 1005 who were offered aHT screening, seven (0.7%) declined, resulting in an acceptance of 998/1005 (99.3%) and an overall screening coverage among eligible participants of 998/1091 (91.5%).

### Initial aHT screening

Of the 998 participants screened, 671 (67.2%) had normal blood pressure values, 206 (20.6%) were already known to have aHT, 4 (0.4%) were newly diagnosed with aHT, and 117 (11.7%) showed elevated readings in the initial screening that necessitated a subsequent confirmatory measurement.

### Confirmatory BP measurement

Of the 117 participants who required a confirmatory BP measurement after initially elevated readings, 5/117 (4.3%) refused confirmatory screening, 50/117 (42.7%) had high normal BP, and 62/117(53.0%) were confirmed to have aHT.

Among the 1091 eligible participants, the CHWs achieved screening coverage of 37.7%, 77.3%, and 91.5% at one, three, and six months, respectively. Overall, 66 (6.0%) were newly found to have aHT.

### Screening and diagnosis of DM

Of the 803 eligible participants, 791 (98.5%) were offered screening for DM between 30 March 2022 and 31 December 2023. 12 were offered screening after the study closure date. Three declined screening, resulting in an acceptance of 788/791 (99.6%) and a screening coverage rate of 788/803 (98.1%).

### Initial DM screening

Of the 788 participants screened, 460 (58.4%) were found to have no DM, 26 (3.3%) were already known to live with DM, three (0.4%) were newly diagnosed with DM, and the remaining 299 (37.9%) showed elevated readings in the initial screening that necessitated confirmation through a subsequent measurement at fasting state.

Of the 299 participants who required a confirmatory measurement, 4 (1.3%) declined, 258 (86.3%) had a normal second reading, 24 (8.0%) had pre-DM, and 13 (4.3%) were newly diagnosed with DM.

Among the 803 participants eligible for DM screening, the CHW achieved the screening coverage of 19.7%, 76.2%, and 92.7% at one, three, and six months, respectively. Overall, 16 (2.0 %) were newly diagnosed with DM.

## Qualitative Results

A total of 10 participants were interviewed to evaluate the acceptability and perceptions of the CHW-led home-based screening and diagnosis for aHT and DM. Thematic analysis identified four major overarching themes: convenience, trust, and satisfaction with CHWs, perceived impact on access to care, and suggestions for service expansion.

### Convenience of home-based services

Participants widely appreciated the convenience of CHW-led services, citing the reduction of travel time, reduced transport costs, and avoidance of long clinic queues. The accessibility of screenings at home allowed individuals, especially those with physical disabilities, to participate in routine health assessments with ease. A 63-year-old male participant highlighted his preference for home-based screening, citing convenience as a key factor. He further elaborated on the challenges he faced when attending hospital appointments, noting that his partial blindness made it difficult for him to navigate unfamiliar environments.

*“Well, I prefer being helped at home because when going to the hospital, I was struggling because am even partially blind.”*

### Trust and satisfaction with CHWs

Community members expressed high levels of trust and satisfaction with CHWs, emphasizing their professionalism, accessibility, and consistency in providing care. The participants appreciated the regular health check-ups and guidance provided by CHWs. This sentiment was echoed by the male participant, who expressed his appreciation to CHW for monitoring their health on a regular basis without going to the health facilities.

*“We were very satisfied by her services, and we would like to have such services all the time so that our health can be frequently checked up. And, that we can know how much weight we have lost or gained as well as knowing how our high blood (pressure) is doing and be updated with our health every time.”*

A 61-year-old woman with aHT expressed satisfaction with receiving health services at home without the need to travel to the health facilities.

*“Well, we prefer here at the village because we really got lucky that she was appointed as the community health worker to deliver services for us here at the village.”*

### Improved access to care

Participants felt that the CHW-led model enhanced access to care by delivering services directly to communities, overcoming geographical and logistical barriers. Participants noted that these services were particularly beneficial for people in remote areas and those unable to travel to health facilities. This was confirmed by a 39-year-old woman, who had never been tested for DM and aHT, and expressed her gratitude to the CHWs.

*“I can generally say the services are quite accessible because the health worker moves around the village providing us with high blood and sugar diabetes services, and if he finds someone with such a disease, he gives them guidance on what should be done as way forward to deal with the disease.”*

### Suggestions for service expansion

Participants recommended broadening the scope of CHW-led services to include additional health needs such as HIV testing, mental health support, and services for individuals with disabilities. Participants believe that including people with disabilities in health services provided by CHW is crucial, given the significant logistical and cost barriers they face in accessing health facilities. In most cases, unlike others, people with disabilities require special transport instead of standard taxis, further increasing their challenges.

*“Besides high blood pressure screening, you can provide us with HIV services, you can provide us with mental health services. We have people who are bed ridden in our village, I don’t know what kind of diseases I can categories them into, so you should also provide services for people with disabilities, the health worker should approach them and see what kind of assistance they need so that health services are brought closer to them too.”*

Some participants also highlighted the need for CHWs to assist with minor injuries and other basic health concerns at the community level. A 37-year-old female believes that the inclusion of other services will be beneficial to the community.

*“The provision of HIV self-testing services at the village level could prove beneficial in addressing the concerns of individuals who are reluctant to visit clinics. By offering these services at the community level, individuals can access them without the need for travel, potentially increasing the uptake of such services. It would be beneficial for the community to utilise the services of the CHW, as she is readily available and frequently in attendance. Additionally, she can assist with the management of minor injuries and wounds, which are often challenging to treat in a remote setting.”*

**Table 1:** Sociodemographic and clinical characteristics of study participants.

	OVERALL POPULATION N (%)
**Sex:**	
Male Female	788 (43.5%) 1023 (56.5%)
**Age**	
Median (IQR) <10 years 10 – 15 years 16 – 39 years ≥ 40 years	24 (11-25.5) 395 (21.8%) 250 (13.8%) 585 (32.3%) 581 (32.1%)
**Relationship status**	
Married/in relationship Single/widowed/divorced No answer <16 years	627 (34.6%) 532 (29.4%) 7 (0.4%) 645 (35.6%)
**Educational status**	
No school Completed primary school Completed secondary school Completed tertiary education <16 years	78 (4.3%) 630 (34.8 %) 403 (22.3%) 55 (3%) 645 (35.6%)
**Employment status**	
Regular income (self-employed/employed) Own plot or livestock No income Full time student/scholar Retired/disabled/sick No answer <16 years	435(24.0%) 357 (19.7%) 134 (7.4%) 48 (2.7%) 52 (2.9%) 140 (7.7%) 645 (35.6%)
**HIV Status**	
Known to live with HIV. Tested HIV negative ≤ 12 month ago Tested HIV negative > 12 months ago No answer Unknown	154 (8.5%) 748 (41.3%) 341 (18.8%) 23 (1.3%) 545 (30.1%)
**Body Mass Index kg/m^2^ among ≥ 16 years**	
Median (IQR) <18.5 18.5 – 24.9 25 −34.9 35 and above <16 years	23.4 (20.3-27.7) 137 (11.8%) 572 (49.1%) 381 (32.7%) 76 (6.5%) 645 (35.6%)
**Previously diagnosed with hypertension (aHT)**	
Yes No Unknown <16 years	373 (20.6 %) 776 (42.8%) 17 (0.9%) 645 (35.6%)
**Taking antihypertensive medicine**	
Yes No Unknown <16 years	223 (12.3%) 930 (51.4%) 13 (0.7%) 645 (35.6%)
**Previously diagnosed with diabetes (DM)**	
Yes No Unknown <16 years	70 (3.9 %) 1074 (59.3%) 22 (1.2%) 645 (35.6%)
**Taking anti-diabetic medication**	
Yes No Unknown <16 years	24 (1.3%) 1124 (62.1%) 18 (1 %) 645 (35.6%)
**Currently Smoking**	
Yes No <10 years	387 (21.4%) 1029 (56.8%) 395 (21.8%)
**Smoking frequency (n=387)**	
1-5 cigarettes per day 6-10 cigarettes per day 11-20 cigarettes per day More than 20 cigarettes per day Other forms of tobacco consumption	196 (50.6%) 32 (8.3%) 11 (2.8%) 5 (1.3%) 143 (37%)
**Self-reported history heart attack**	
Yes No Unknown <16 years	78 (4.3%) 1081 (59.7%) 7 (0.4%) 645 (35.6%)
**Self-reported history of stroke**	
Yes No Unknown <16 years	17 (1%) 1138 (62.8%) 11 (0.6%) 645 (35,6%)
**Close relative previously diagnosed with DM**	
Yes No Unknown <16 years	98 (5.4%) 1007 (55.6%) 61 (3.4%) 645(35.6%)

*Notes:* < 16 years: Children and adolescents under 16 were not asked the questions.

<10 years: Children and adolescents under 10 were not asked the questions.

## Discussion

We conducted a mixed-methods pilot study to assess the feasibility, acceptability, and uptake of CHW-led aHT and DM screening and diagnosis in 10 villages in Lesotho. The study demonstrated that this approach is both feasible and highly acceptable, achieving over 95% screening coverage within six months. Participants highlighted improved access to care, convenience, trust, and satisfaction with CHW as main reasons for the positive perception of the CHW’s services. Participants recommended exploring the expansion of CHW-led services to address other health needs, such as HIV testing, mental health support, and care for individuals with disabilities as identified in the qualitative interviews. These community-driven suggestions highlight opportunities for future research to assess the feasibility and impact of broader CHW-led health interventions.

Although screening coverage was high, the proportion of new diagnoses of aHT and DM was relatively low. This may reflect a high level of awareness among participants of their existing conditions, possibly due to previous primary health campaigns, improved access to care, or the availability of free health services in Lesotho. In a recently conducted population-based survey in Lesotho, 69.7% of participants with aHT and 48.4% of those with DM were aware of their condition [31].

The feasibility of CHW-led home-based service delivery is demonstrated by the high proportion of household heads and community members who consented to home-based screening and diagnosis of aHT and DM. This indicates significant potential for CHWs to effectively deliver home-based services for NCDs.

Participants expressed satisfaction with home-based care, which reduced logistical barriers such as travel time, transportation costs, and long queues at clinics. As one participant noted, the ability to receive care at home was particularly beneficial for individuals with physical disabilities. These findings align with findings from a South African study where home-based tuberculosis screening by CHWs was appreciated for its accessibility and eliminating the need for multiple clinic visits and ensuring prompt access to reliable information [46]. Some studies highlight crucial elements from the client’s perspective that influence the acceptability and feasibility of healthcare services. These factors are shaped by the content, context, and quality of care, which, in turn, are influenced by clients’ attitudes toward the services and their perceptions of acceptability and feasibility before participation [47, 48]. Key factors include the service’s relevance to the clinical issue, alignment with the service user’s lifestyle, convenience, overall satisfaction and perceived effectiveness of the care provided [49, 50].

Previous studies demonstrated CHWs’ effectiveness in delivering preventive and health promotion services, particularly in HIV, tuberculosis, and maternal and child health [20, 51, 52]. CHW-delivered preventive services are considered cost-effective [53, 54]. However, limited data exists from Africa regarding CHWs-led screening and diagnosis of aHT and DM at household level [55, 56]. To our knowledge, this study is the first to investigate the feasibility and acceptability of CHW-led aHT and DM screening and diagnosis supported by a tablet-based clinical decision support system and without direct health professional supervision.

In summary, our findings suggest that screening and diagnosis for aHT and DM by CHWs who are assisted by a tablet-based clinical decision support system is acceptable and feasible. Further, CHW-led screening and diagnosis may be a promising approach to increase diagnostic coverage for aHT and DM, particularly among populations whose access to clinic-based screening and diagnosis may be difficult due to long travel distance, financial constraints, physical impairment, or personal perception of clinic-based care. Our findings may encourage further larger-scale implementation studies on clinical decision support system assisted by CHW-led NCD screening in similar settings.

### Strengths and limitations

This study has several strengths. First, the use of validated diagnostic criteria and a clinical decision support system ensured accurate and scalable CHW-led screening. Second, adherence to local and international guidelines strengthened the reliability of diagnoses. Third, the mixed-methods approach provided both quantitative and qualitative insights, enhancing the understanding of feasibility and acceptability. Last, the high screening coverage (>90%) demonstrated the potential of CHW-led interventions in rural settings.

However, there are limitations. First, the study was conducted in only 10 villages, limiting generalizability. Second, there is a potential for selection bias when village leaders are involved in identifying CHW candidates. Third, while feasibility and acceptability were assessed, long-term clinical outcomes remain unknown. Fourth, reliance on CHWs for data collection, despite training, may introduce measurement inconsistencies. Last, the qualitative sample was small, capturing only a limited range of community perspectives. Future studies should assess sustainability and health impact on a larger scale.

## Conclusion

This study highlights the feasibility and acceptability of CHW-led home-based screening and diagnosis for aHT and DM in rural Lesotho. The high uptake underscores the potential of CHW-led interventions to help close the diagnostic gap for NCDs, particularly in areas with limited healthcare access.

By integrating CHWs with clinical decision support systems, this model presents a promising strategy to enhance early detection of chronic diseases in remote areas in low-income countries. Future research should focus on its scalability and long-term sustainability to inform national health policies and broader implementation efforts.

## Data Availability

The datasets used and analyzed during the pilot study are available from the corresponding author on request.
